# Emergency surgical management of acute Stanford type A aortic dissection in a primigravida at 29 weeks gestation: A case report

**DOI:** 10.1097/MD.0000000000050813

**Published:** 2026-09-18

**Authors:** Jianping Gan, Jingjing An

**Affiliations:** a Operating Room, Department of Anesthesiology, West China Hospital, Sichuan University/West China School of Nursing, Sichuan University, Chengdu, Sichuan, China.

**Keywords:** Bakri balloon, patient blood management, pregnancy, Stanford type A aortic dissection, total arch replacement

## Abstract

**Rationale:**

Acute aortic dissection during the 3rd trimester of pregnancy is a catastrophic cardiovascular emergency. The management is complex, requiring a multidisciplinary strategy to balance the competing risks of aortic rupture and fetal prematurity, as well as the conflict between surgical anticoagulation and postpartum hemorrhage.

**Patient concerns:**

We report a case of a 34-year-old primigravida (29 weeks and 3 days of gestation) presenting with sudden, severe chest and back pain lasting over 7 hours following physical exertion. She had a history of developmental dysplasia of the left hip and thalassemia.

**Diagnoses:**

Computed tomography angiography confirmed acute Stanford type A aortic dissection involving the ascending aorta and aortic arch branches and extending to the bilateral common iliac arteries, with severe true-lumen compression.

**Interventions:**

Given the life-threatening nature of the dissection and fetal viability, a multidisciplinary team performed an emergency coordinated sequential combined procedure. Cesarean delivery was undertaken 1st. Because of initial uterine atony and the requirement for subsequent full systemic heparinization for cardiopulmonary bypass, a Bakri intrauterine balloon was inserted to secure uterine hemostasis. After abdominal closure, the patient underwent aortic valve commissural resuspension, total arch replacement, and frozen elephant trunk implantation.

**Outcomes:**

A viable preterm female infant weighing 1320 g was delivered and survived to neonatal intensive care unit discharge after respiratory support. Maternal uterine hemostasis was maintained during systemic heparinization, with estimated surgical blood loss of approximately 600 mL. Despite the patient’s underlying thalassemia, no allogeneic red blood cell transfusion was required; transfusion was limited to 1 therapeutic unit of irradiated apheresis platelets. The mother recovered without neurological deficits.

**Lessons:**

This case illustrates a coordinated cesarean-delivery-followed-by-aortic-repair strategy that avoided fetal exposure to cardiopulmonary bypass. Bakri balloon tamponade provided an organ-sparing means of securing uterine hemostasis before systemic heparinization. In young patients with thoracic aortic disease and possible syndromic features, structured genetic evaluation and family surveillance may be considered when feasible.

## 1. Introduction

Acute aortic dissection (AAD) is a rare but life-threatening complication of pregnancy and occurs most frequently in the 3rd trimester or early postpartum period because of increased hemodynamic stress and pregnancy-related changes in the aortic wall. Prompt diagnosis and surgery are essential for acute type A dissection.^[1]^ Contemporary ACC/AHA and ESC guidance emphasizes multidisciplinary management and individualized decisions based on maternal stability, gestational age, fetal viability, and aortic anatomy.^[1,2]^ We present a 34-year-old primigravida with Stanford type A AAD, thalassemia, and developmental dysplasia of the left hip who was treated with cesarean delivery followed by extensive aortic reconstruction. A Bakri balloon was used to manage the conflict between obstetric hemostasis and systemic anticoagulation.

## 2. Case report

A 34-year-old female, gravida 1, para 0, at 29 weeks and 3 days of gestation, presented to our emergency department with sudden-onset, continuous tearing pain in the chest and back lasting over 7 hours. The pain began after physical exertion and was accompanied by dizziness, palpitations, and generalized weakness. Her medical history included genetically confirmed thalassemia, coexisting iron-deficiency anemia, and developmental dysplasia of the left hip. Before admission, she had received oral polysaccharide iron complex and folic acid. On admission, her hemoglobin level was 121 g/L. Family history was notable for her father’s death from a reported acute myocardial infarction; however, no confirmed family history of thoracic aortic disease was available.

**Figure 1.**
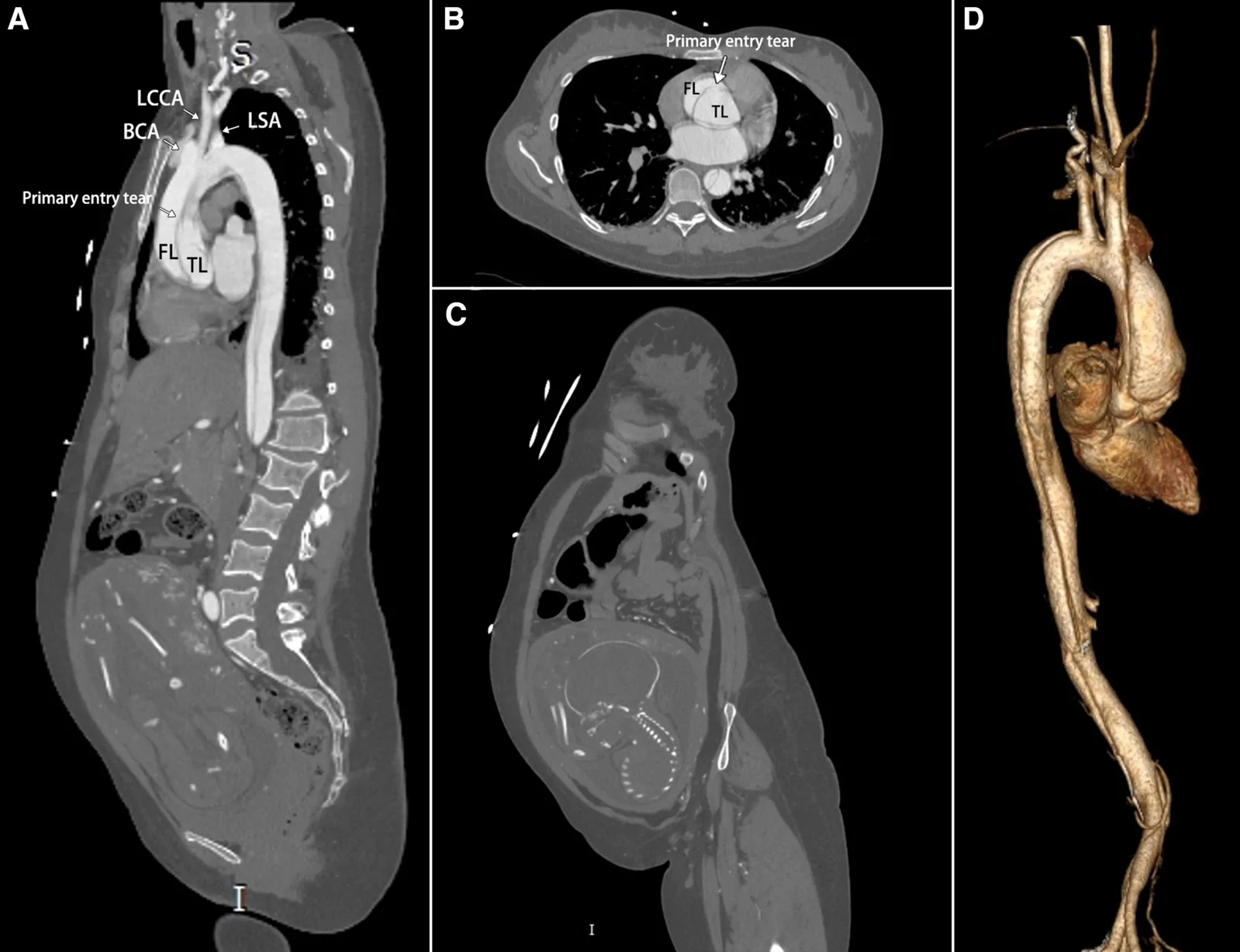
Preoperative computed tomography angiography (CTA) findings in the pregnant patient. (A) Sagittal reformatted CTA image demonstrating acute Stanford type A aortic dissection involving the ascending aorta and aortic arch. The true lumen (TL), false lumen (FL), brachiocephalic artery (BCA), left common carotid artery (LCCA), and left subclavian artery (LSA) are indicated. (B) Axial CTA image showing the TL and FL in the ascending aorta. The arrows in panels (A and B) indicate the site corresponding to the primary entry tear at the aortic sinus, as confirmed intraoperatively. (C) Sagittal CTA image showing the viable fetus in utero at 29 weeks and 3 days of gestation. (D) Three-dimensional volume-rendered CTA reconstruction demonstrating the extent of the aortic dissection. BCA = brachiocephalic artery, FL = false lumen, LCCA = left common carotid artery, LSA = left subclavian artery, TL = true lumen.

Upon admission, emergency computed tomography angiography (CTA) demonstrated Stanford type A aortic dissection involving the ascending aorta, aortic arch branches, and bilateral common iliac arteries (Fig. 1). The brachiocephalic, left common carotid, and left subclavian arteries were involved, and the true lumen was severely compressed. Transthoracic echocardiography performed approximately 7 weeks before admission showed an aortic root diameter of 38 mm, measured at the sinuses of Valsalva in the parasternal long-axis view at end-diastole using the leading-edge-to-leading-edge convention.^[1]^ Indexed to a body surface area of 1.69 m^2^, this corresponded to an aortic size index of 2.25 cm/m^2^. CTA also showed left hip dislocation with associated muscle atrophy.

General anesthesia was induced with etomidate 12 mg, fentanyl 250 μg, rocuronium 70 mg, and an initial dose of sufentanil 20 μg. During subsequent maintenance, propofol was infused at approximately 6 mg/kg/h and remifentanil at approximately 0.25 μg/kg/min. Sevoflurane, at a recorded concentration of approximately 2%, was introduced later, after fetal and placental delivery, during the cardiac surgical phase. Additional neuromuscular blockade comprised rocuronium 30 mg and 3 4-mg doses of vecuronium. Before cardiopulmonary bypass (CPB), continuous infusions of nitroglycerin at 0.1 mg/h and nicardipine at 2.5 mg/h, together with intermittent esmolol and additional nicardipine, were used to maintain maternal mean arterial pressure at 60 to 70 mm Hg.

**Figure 2.**
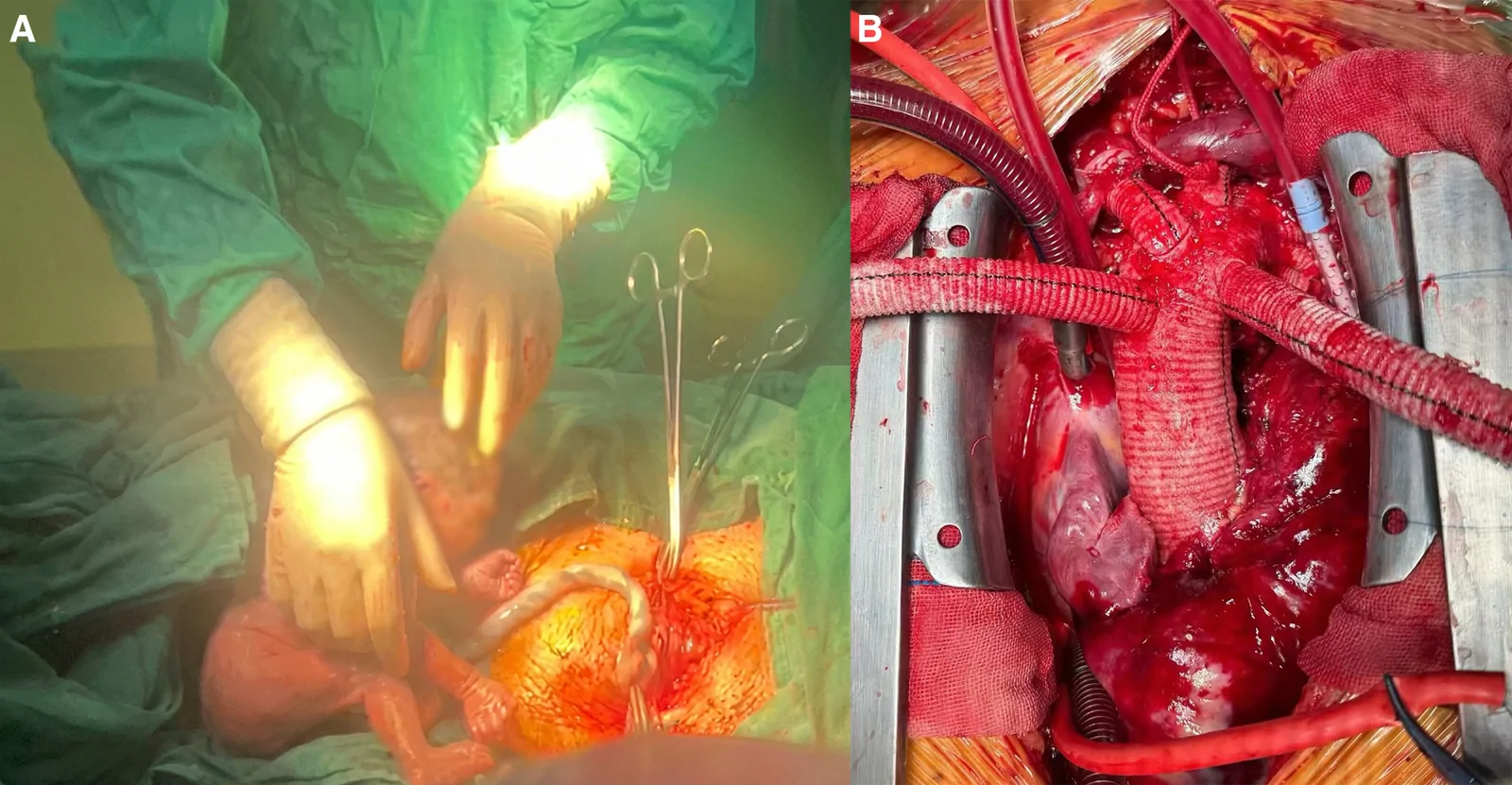
Intraoperative views during the combined procedures. (A) The premature female infant delivered via emergency cesarean section. (B) The vascular anastomosis of the 4-branched vascular graft during total aortic arch replacement.

A lower-segment transverse cesarean section was performed 1st. A live female infant weighing 1320 g was delivered in breech presentation, with Apgar scores of 5, 8, and 8 (Fig. 2A). Following placental delivery, uterine atony with active bleeding was initially treated with uterine massage, intravenous carbetocin 100 μg, and intravenous tranexamic acid 1 g. Neither methylergonovine nor carboprost was administered. Because subsequent aortic repair required full systemic heparinization for CPB, secure uterine hemostasis was essential. A Bakri balloon was therefore inserted under ultrasound guidance, inflated with 150 mL saline, and secured with vaginal gauze packing before systemic heparinization.

Following abdominal closure, the cardiac team proceeded via median sternotomy. Intraoperative inspection localized the primary intimal tear to the aortic sinus, with the dissection extending into the arch branches and associated hemopericardium. Full systemic heparinization was achieved with 23,500 U of heparin, and CPB was established through right femoral arterial cannulation. Total CPB duration was 212 minutes, and the aortic cross-clamp time was 159 minutes. Lower-body hypothermic circulatory arrest was maintained for 20 minutes at a minimum core temperature of 24°C, corresponding to low-moderate hypothermia rather than deep hypothermia.^[3]^ During the same interval, bilateral selective antegrade cerebral perfusion was delivered through the brachiocephalic and left common carotid arteries at a total bilateral flow rate of 6 mL/kg/min. Both nasopharyngeal and bladder temperatures reached a minimum of 24°C. The repair comprised aortic valve commissural resuspension, deployment of a 24 × 120 mm frozen elephant trunk stent (MicroPort) into the descending aorta, and total arch replacement with a 4-branched vascular graft (Maquet). The graft was anastomosed to the frozen elephant trunk stent and the arch vessels (Fig. 2B). The patient was successfully weaned from CPB, after which 180 mg protamine was administered. The Bakri balloon remained in situ throughout systemic heparinization. Estimated surgical blood loss was approximately 600 mL. Allogeneic transfusion was limited to 1 therapeutic unit of irradiated apheresis platelets. Despite the patient’s underlying thalassemia, no allogeneic red blood cell transfusion was required.

**Figure 3.**
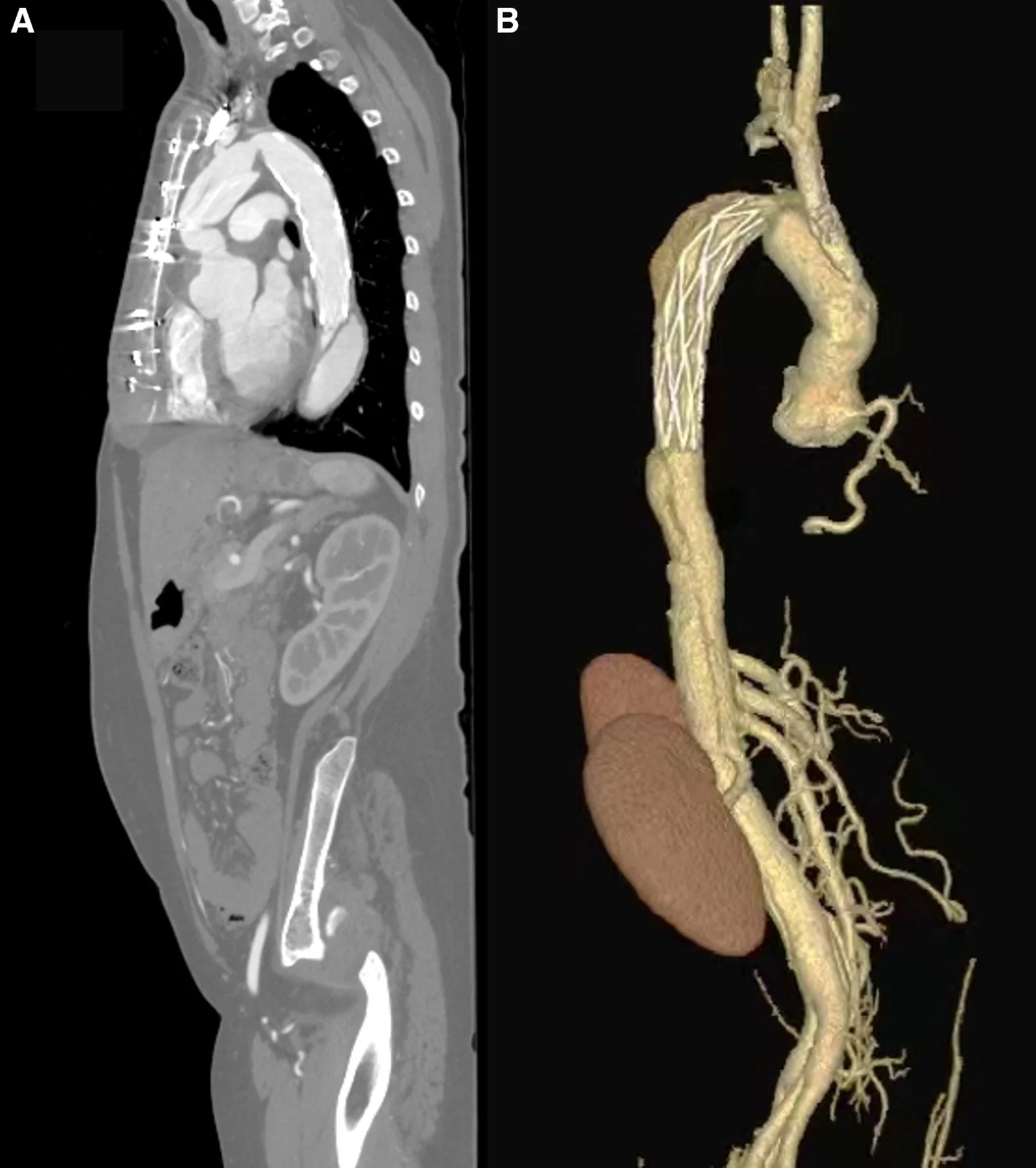
Postoperative computed tomography angiography (CTA) at 1-month follow-up. (A) CTA image obtained 1 month postoperatively, showing intact anastomoses without any signs of endoleak or pseudoaneurysm. (B) Three-dimensional (3D) reconstruction of the aorta confirming excellent patency of the graft and the reconstructed arch vessels.

The maternal postoperative course was uncomplicated, and the patient was extubated after hemodynamic stabilization. Approximately 24 hours after insertion, the Bakri balloon was removed using a staged deflation protocol. First, 75 mL was aspirated, followed by 30 minutes of observation. In the absence of recurrent bleeding, the remaining 75 mL was aspirated, after which the vaginal packing and fully deflated balloon were removed. The patient was discharged on postoperative day 10. At the 1-month follow-up, CTA demonstrated intact anastomoses and patent reconstructed arch vessels (Fig. 3).

Two minutes after birth, poractant alfa (Curosurf; Chiesi Farmaceutici) was instilled via the endotracheal tube at a total dose of 240 mg (approximately 182 mg/kg). The infant subsequently received invasive mechanical ventilation for approximately 4 days and 15 hours, followed by noninvasive respiratory support until postnatal day 29. Serial cranial ultrasonography showed no definite evidence of germinal matrix hemorrhage or intraventricular hemorrhage. At discharge from the neonatal intensive care unit on postnatal day 45, corresponding to a postmenstrual age of 35 weeks and 6 days, the infant weighed 2140 g and tolerated 40 mL per feed every 3 hours without vomiting or abdominal distension. Neurologic examination at discharge showed normal limb tone, elicitable primitive reflexes, and some head-righting response. The key clinical milestones from symptom onset through maternal and neonatal discharge are summarized in Table 1.

**Table 1:** Clinical timeline.

Clinical milestone	Recorded time	Interval or detail
Symptom onset	06:20	Sudden tearing chest and back pain
Emergency department presentation	13:21	7 h 1 min after symptom onset
CTA diagnostic confirmation	14:28	CTA images permitted immediate diagnosis
Emergency operation requested	16:28	Decision for emergency surgery
Skin incision	19:22	174 min after operative request
Cesarean delivery	19:25	3 min after skin incision
Placental delivery	19:28	Reference point for heparin interval
Bakri balloon placement	19:45	150 mL saline with vaginal gauze packing; 17 min after placental delivery
Full systemic heparinization	22:14	23,500 U; 166 min after placental delivery
CPB initiation	22:31	17 min after heparin; right femoral arterial cannulation
Lower-body HCA and bilateral SACP	00:44–01:04	20 min; total bilateral SACP flow 6 mL/kg/min; minimum nasopharyngeal and bladder temperatures 24°C; low-moderate hypothermia
CPB completion	02:03	CPB initiated at 22:31; total duration 212 min
Protamine administration	02:09	180 mg; 6 min after calculated CPB completion
Bakri balloon removal	~24 h after insertion	Staged deflation: 75 mL, observation for 30 min, then remaining 75 mL; packing removed with balloon
Maternal hospital discharge	Postoperative day 10	Clinically stable
Neonatal respiratory course	Birth to postnatal day 29	Invasive support for 4 d 14 h 54 min, followed by noninvasive support
NICU discharge	Postnatal day 45	Weight 2140 g; postmenstrual age 35 weeks and 6 days

CPB = cardiopulmonary bypass, CTA = computed tomography angiography, HCA = hypothermic circulatory arrest, NICU = neonatal intensive care unit, SACP = selective antegrade cerebral perfusion.

## 3. Discussion

AAD during pregnancy is a rare but catastrophic event associated with substantial maternal and fetal risks. Its incidence is increased during the 3rd trimester and postpartum period.^[2]^ This pregnancy-related susceptibility likely reflects a combination of hormonal and hemodynamic changes, including expanded intravascular volume and increased cardiac workload. Estrogen and progesterone may contribute to structural remodeling of the aortic media and weakening of the vessel wall.^[4]^ In the present case, physical exertion immediately preceded the characteristic chest and back pain; however, this temporal association does not establish causality.

The occurrence of extensive aortic dissection in a young patient raises the possibility of heritable thoracic aortic disease. The aortic root diameter of 38 mm and history of developmental dysplasia of the hip represent additional but nonspecific findings and do not establish a diagnosis of Marfan or Loeys–Dietz syndrome. After discharge, referral to a multidisciplinary aortopathy clinic, genetic counseling, and structured genetic evaluation may be considered when feasible. If pursued, a clinically validated next-generation sequencing panel would typically include established genes associated with thoracic aortic aneurysm and dissection, such as FBN1, TGFBR1, TGFBR2, SMAD3, TGFB2, TGFB3, COL3A1, ACTA2, and MYH11.^[1]^ Identification of a pathogenic or likely pathogenic variant could guide gene-specific management and cascade testing of the child and 1st-degree relatives. If testing is negative or yields only a variant of uncertain significance, screening aortic imaging of 1st-degree relatives may still be considered.^[1]^ Long-term surveillance of the residual aorta is warranted, with the imaging interval individualized by a multidisciplinary aortic team.

Management of AAD during pregnancy requires balancing maternal survival with fetal viability. The optimal strategy should be individualized according to maternal clinical stability, gestational age, fetal viability, and aortic anatomy. At earlier gestational ages, particularly before approximately 28 weeks, maternal aortic repair with the fetus in utero may be considered; however, fetal mortality during CPB remains substantial, owing in part to hypothermia and nonpulsatile flow.^[5,6]^ In the present case, the fetus was considered viable at 29 weeks and 3 days; therefore, we adopted a cesarean-delivery-followed-by-aortic-repair strategy. This strategy avoided fetal exposure to CPB, eliminated the hemodynamic stress of labor, reduced intra-abdominal pressure for surgical exposure, and facilitated subsequent maternal resuscitation and aortic repair.

A critical challenge in such combined procedures is the conflict between systemic heparinization for CPB and the risk of postpartum hemorrhage, particularly in the presence of uterine atony. In this case, initial uterine atony was managed with uterine massage, carbetocin, tranexamic acid, and Bakri balloon tamponade before systemic heparinization. Unlike hysterectomy, the balloon provides organ-sparing mechanical compression. Because strict anti-impulse control was essential, methylergonovine was avoided owing to its potential to cause abrupt hypertension and coronary vasospasm. Carboprost was withheld because its bronchoconstrictive and vascular effects could impose additional cardiopulmonary stress.^[7]^ With the Bakri balloon in place, uterine hemostasis was maintained throughout systemic heparinization. The overall patient blood management strategy was further supported by a well-preserved preoperative hemoglobin level following antepartum iron and folate supplementation, limited platelet transfusion, and avoidance of allogeneic red blood cell transfusion. The favorable outcome illustrates the feasibility of this approach in the present patient but does not establish its superiority over alternative hemostatic strategies.^[8]^

Given the extensive involvement of the aortic arch and descending aorta, total arch replacement combined with frozen elephant trunk implantation was selected. This strategy addressed both the arch and proximal descending aortic disease. The stented segment expanded the true lumen and may promote false-lumen thrombosis and favorable distal aortic remodeling, potentially reducing the risk of late aneurysmal degeneration and distal re-intervention.^[9]^

This case report has several limitations. First, definitive genetic testing for heritable thoracic aortic disease was not available, precluding confirmation of a specific genetic diagnosis. Second, as a single case, this report cannot establish the efficacy or superiority of Bakri balloon tamponade in this setting. Finally, the relatively short maternal and neonatal follow-up precluded assessment of long-term aortic remodeling and neurodevelopmental outcomes.

## 4. Conclusion

This case illustrates the feasibility of an emergency, multidisciplinary, sequential surgical strategy consisting of cesarean delivery followed by aortic repair for acute Stanford type A aortic dissection at 29 weeks and 3 days of gestation. In this patient, Bakri balloon tamponade helped maintain uterine hemostasis during subsequent systemic heparinization for cardiopulmonary bypass.

## Acknowledgments

During the preparation of this manuscript, the authors used ChatGPT (OpenAI) solely for English language polishing and proofreading. The authors wrote the original content, thoroughly reviewed and edited all AI-generated suggestions, and take full responsibility for the final content of the publication.

## Author contributions

**Writing – original draft:** Jianping Gan.

**Writing – review & editing:** Jingjing An.
